# Supplementary material for: Budget impact analysis of HARMONIC FOCUS™+ Shears for mastectomy and breast-conserving surgery with axillary lymph node dissection compared with monopolar electrocautery from an Italian hospital perspective
Source: PLoS One. 2022 Jun 21;17(6):e0268708. doi: 10.1371/journal.pone.0268708 (PMC9212163; doi:10.1371/journal.pone.0268708)
Supplement: S1 Table — Abbreviations: DSA: deterministic sensitivity analysis; EUR: Euros; LOS: length of stay; OR: operating room; RR: risk ratio; PSA: probabilistic sensitivity analysis. (DOCX) [file pone.0268708.s001.docx]

SUPPORTING INFORMATION

S1 Table. Parameters and distributions applied in the sensitivity analyses.

| **DSA/PSA parameter** | **Primary analysis value** | **Lower bound** | **Upper bound** | **PSA Distribution** |
| --- | --- | --- | --- | --- |
| Cost monopolar electrocautery, EUR | 118.99 | 89.24 | 148.73 | Gamma |
| Cost of HARMONIC FOCUS™+, EUR | 551.67 | 413.75 | 689.59 | Gamma |
| OR time with monopolar electrocautery, mins | 120.00 | 90.00 | 150.00 | Gamma |
| OR time with HARMONIC FOCUS™+, mins | 115.00 | 86.25 | 143.75 | Gamma |
| OR time cost per minute, EUR | 7.03 | 5.27 | 8.79 | Gamma |
| LOS with monopolar electrocautery, days | 5.00 | 3.75 | 6.25 | Gamma |
| LOS with HARMONIC FOCUS™+, days | 3.00 | 2.25 | 3.75 | Gamma |
| Hospital stay cost per day, EUR | 655.27 | 491.45 | 819.08 | Gamma |
| Seroma baseline rate with monopolar electrocautery, % | 0.30 | 0.23 | 0.38 | Beta |
| Seroma reduction rate, Mantel-Haenszel RR | 0.11 | 0.01 | 0.82 | Log-normal |
| Seroma incremental cost per episode, EUR | 7.11 | 5.33 | 8.89 | Gamma |
| Postoperative drain duration with monopolar electrocautery, days | 5.00 | 3.75 | 6.25 | Gamma |
| Postoperative drain duration with HARMONIC FOCUS™+, days | 3.00 | 2.25 | 3.75 | Gamma |
| Postoperative drainage cost per patient per day, EUR | 42.77 | 32.08 | 53.46 | Gamma |

**Abbreviations:** DSA: deterministic sensitivity analysis; EUR: Euros; LOS: length of stay; OR: operating room; RR: risk ratio; PSA: probabilistic sensitivity analysis.
